# Factors Associated with Self and Informant Ratings of the Quality of Life of People with Dementia Living in Care Facilities: A Cross Sectional Study

**DOI:** 10.1371/journal.pone.0015621

**Published:** 2010-12-13

**Authors:** Christopher Beer, Leon Flicker, Barbara Horner, Nick Bretland, Samuel Scherer, Nicola T. Lautenschlager, Frank Schaper, Osvaldo P. Almeida

**Affiliations:** 1 WA Centre for Health and Ageing, Centre for Medical Research, Perth, Western Australia, Australia; 2 School of Medicine and Pharmacology, University of Western Australia, Crawley, Western Australia, Australia; 3 Centre for Research on Ageing, Curtin University of Technology, Perth, Western Australia, Australia; 4 Rowethorpe Medical Centre, Bentley, Western Australia, Australia; 5 Royal Freemasons Homes of Victoria, Prahran, Victoria, Australia; 6 Academic Unit for Psychiatry of Old Age, St Vincent's Health, Department of Psychiatry, University of Melbourne, Parkville, Victoria, Australia; 7 School of Psychiatry and Neurosciences, University of Western Australia, Australia; 8 Alzheimer's Australia WA Ltd, Perth, Western Australia, Australia; University of Valencia, Spain

## Abstract

**Background:**

There is no consensus regarding the optimal approach to assessment of the quality of life of people with dementia. We undertook the present study to describe and determine the factors associated with ratings of the quality of life of a cohort of people with dementia living in a residential care facility.

**Methodology/Principal Findings:**

351 people with dementia living in residential care facilities, and their staff and family informants participated in this cross sectional observational study. Quality of life was measured using self (Quality of Life in Alzheimer's Disease [QoL-AD] scale), and informant (QoL-AD and Alzheimer's Disease Related QoL Scale) reports. 226 people (64%) with dementia (median MMSE 17; 12–21) were able to self rate the QoL-AD scale and these subjects' ratings were compared to ratings by staff and family. Both staff and family informant ratings of the QoL-AD underestimated self ratings (mean difference −7.8, 95% CI −8.8, −6.7 for staff rated QoL-AD; and mean difference −7.2, 95% CI −8.5, −6.0 for family rated QoL-AD). Self ratings of QoL were lower among people who were restrained, had fallen or had pain. Informant ratings of the QoL of the participants with dementia were consistently and significantly lower for people with severe cognitive impairment, who had fallen, had presence of neuropsychiatric symptoms, or where care giver distress was present. Documented restraint, reported pain and neuropsychiatric symptoms were independently associated with lower self rating of the QoL-AD in multivariate models. Cognitive impairment, case conferencing, hospitalizations and neuropsychiatric symptoms were found to be independently associated with staff rated ADRQL.

**Conclusions:**

The majority of people with dementia living in residential care facilities can rate their own QoL. Informant ratings underestimate self ratings of QoL of people with dementia, and appear to be associated with factors which are not associated with self ratings.

## Introduction

Moderate to severe dementia is common among people requiring residential care. This population generally has high levels of impairment and complex care needs. Currently available data suggest that people with moderate to severe dementia frequently experience distressing emotions. This may relate to preserved awareness of their deficits, environment and unmet needs.[1]–[2] A person centred[3] approach to care is, therefore, recommended as a way to understand the needs of older adults with dementia. In this context, the quality of life (QoL) of people with dementia (PWD) is an important outcome of care.[4]–[5]

There is evidence that even people with moderate to severe dementia can reliably rate their own quality of life.[6] However, use of staff or family carer informant ratings of quality of life is widespread, particularly when very severe dementia makes reliable self-rating difficult. For PWD living in residential care facilities (RCF), there are potential problems with the use of staff informant ratings. High staff turnover may undermine consistent assignment of staff and preclude reliable estimation of the quality of life of residents. Furthermore, informant ratings may be influenced by specific factors such as the person's severity of cognitive impairment and dependency, the rater's own beliefs regarding dementia and dementia care, and the quality of the relationship between the informant and the person with dementia.[7]–[8] These factors may influence informant ratings of quality of life to a greater extent than individuals' self-ratings.[8] Although these potential problems are recognized, there is a paucity of data assessing ratings of QoL made by family carers of PWD living in RCF. In some cases family carers may be able to provide greater consistency of rating than paid carers, and may have greater insights into the personal perspective of the person with dementia. Moreover, the perspective of family carers regarding the QoL of PWD is of critical importance when clinical decisions are made on behalf of patients.

We undertook the present study to describe and determine the factors associated with, self, staff and family carer informant ratings of the QoL of a cohort of PWD living in RCF.

## Methods

### Ethics Statement

The research was approved by the Human Research Ethics Committee of the University of Western Australia. All participants provided informed consent. For PWD a structured consent procedure was utilized (comprising informed written or verbal consent, as well as the agreement of next of kin) and, when severity of dementia precluded the resident from providing consent that was clearly informed, agreement from the resident's ‘next of kin’.

### Study design

Cross-sectional study

### Setting

Participants were recruited from residential aged care facilities (RCF) in Perth, Western Australia.

### Participants

351 PWD living in residential care facilities (PWD-RCF), their family carers and staff informants participated in the present baseline data collection of the DIRECT study, a randomised controlled trial of educational interventions aiming to improve the QoL of PWD living in RCF (the protocol details have been described elsewhere[9]). In brief, RCF participants were all permanent residents of aged care facilities aged over 65 years, with a clinical diagnosis of dementia and a Mini-Mental State Examination (MMSE) total score lower than or equal to 24. Residents were excluded if the facility staff identified them as being acutely medically unstable or suffering from delirium, or if they were in the terminal stages of a co-morbid illness (e.g. metastatic cancer). The descriptor “family” was used in a general sense and comprised relatives, spouses or partners, and other close personal contacts. Family informants for PWD living in RCF were required to have visited the PWD on average at least once per week over the previous year. Staff informants were required to have known the resident for at least two weeks, and to have observed that resident at least 10 times, or for a minimum of one hour in total, during the previous two weeks.

### Outcome measurements

We assessed QoL comprehensively by using two measures with different characteristics, and utilising multiple perspectives. The Quality of Life in Alzheimer's Disease (QoL-AD) scale was used to measure QoL by self and informant reports.[5], [10] The 13 item QoL-AD scale is a widely used, brief (generally administered in ∼10 min), dementia-specific tool. Available data suggest that the QoL-AD has good inter-rater and test–retest reliability[10]–[11] and can be used to reliably assess the quality of life of people with severe dementia.[6] The QoL-AD has been modified to produce a 15-item scale (maximum score 60) to assess the QoL of PWD living in RCF according to a standard set of instructions.[5] [12]–[13] Informants who could not be interviewed face-to-face were interviewed over the telephone. In face-to-face interviews, participants were handed their own copy of the questionnaire that they could follow, if able to. Participants were able to indicate responses verbally or by circling the response. If a participant was unable to offer responses to more than two items, they were considered unable to complete the measure and their results were excluded from the analyses. The inter-rater reliability of the QoL-AD administered by our research staff has been described previously.[14] In addition, the Alzheimer's Disease Related QoL Scale (ADRQL)[15] was administered to family and staff informants. The ADRQL is a 47 item scale covering 5 domains (social interaction, awareness of self, feelings and mood, enjoyment of activities, and response to surroundings). The ADRQL was administered as a standardized, structured interview over 10–15 minutes. Response items are weighted, producing a maximum score of 100. For both the ADRQL and the QoL-AD higher scores indicate better QoL.

### Predictor variables

Date of birth and gender of participants were recorded, as well as whether the perimeter of the facility was secured. The Standardised Mini-mental State Examination (MMSE),[16] a self report pain scale (Brief Pain Inventory modified verbal form[17]), an observational pain scale (PAIN-AD[18]), and the Neuropsychiatric Inventory- NH version (NPI-NH)[19] were administered by research staff. Research staff recorded whether physical restraints were applied to the resident. This included fixed tray tables, “fall out” chairs and zipped bedding, as well as overt restraints. Research staff audited participants' clinical records, noting reviews by the resident general practitioner in the last month, Comprehensive Medical Assessments (CMA) documented within 6 weeks of admission to the facility, case conferences held in the last month, pain assessment documented in the last 12 months, restraint documented in the last two weeks, presentations to hospital documented within the last month, and documentation of falls occurring in the preceding month. Medications (including as required medications) were counted. The resident's most recent weight, and any previous weight documented in the last 6 months were recorded.

### Data Handling and Analysis

Data were handled using SPSS and Stata (version 11, StataCorp, College Station, Texas). First, descriptive statistics and differences in QoL ratings between pairs of raters were calculated. To determine the significance and magnitude of the influence of predictor variables on QoL ratings by patients, family informants and RCF further analyses were conducted on the subset of data from people able to self rate. We calculated the mean difference, between PWD able to self rate QoL with, and without, each candidate predictor variable and the respective 95% confidence interval (95%CI) for the difference. “Pain reported” was defined as a positive response to the first question in the Brief Pain Inventory (“*Throughout our lives, most of us have had minor aches and pains from time to time. Have you had pain, other than these everyday kinds of pain, today?*”), “Pain observed” was defined as a score of greater than one on the PAIN-AD scale. “Increasing weight” was defined as most recent weight greater than previous weight recording in the last 6 months. Presence of “neuropsychiatric symptoms” was defined as NPI-NH score >14 and “staff distress” as a care giver distress score>4 on the NPI-NH. Other non-categorical predictor variables were categorized at a clinically accepted point (eg MMSE <10). Finally, to determine whether associations between predictor variables and quality of life rating were independent of each other we constructed parsimonious regression models, for both self rating of the QoL-AD, and staff informant rating of the ADRQL. This commenced with a series of univariate models (in which continuous variables were evaluated as such) to identify all candidate predictor variables. Variables with p<0.1 were then included in a multivariate regression model and removed using a backward stepwise process if their significance was not retained, until all remaining variables had p<0.05. We set alpha at 5% and all p-values reported are two-sided.

## Results

The 351 participants had average age of 85.3±7.9 years and a median MMSE score of 14 (interquartile range, IQR 6–19). Approximately two-thirds (64%) of PWD rated their own QoL using the QoL-AD scale. People able to self-rate the QoL-AD had higher MMSE (median 17; IQR 12–21) compared with people who were not able to self-rate the QoL-AD (median 5; IQR 0–11; p<0.001). We obtained staff informant ratings for most PWD (92% using the QoL-AD and 99% using the ADRQL; Table 1). There were few cases where family ratings were the only informant rating available (8 [2.2%] for the QoL-AD and 2 [0.0%] for the ADRQL.

**Table 1:** Self and Informant Ratings of QOL of PWD living in RCF.

Scale and Rater	n (%)351	Mean score*± SD	Mean difference (95% CI)+, n pairs with data	Pearson Correlation+
QOL-ADSelf rated	226 (64%)	41.5+5.9		
Staff rated	324 (92%)	32.1+7.4	−7.8 (−8.8, −6.7), 208 pairs	0.303 (p<0.001)
NOK rated	292 (83%)	32.4+8.2	−7.2 (−8.5, −6.0), 189 pairs	0.309 (p<0.001)
ADRQLStaff rated	347 (99%)	72.8+16.3		
NOK rated	298 (85%)	74.9+14.7	1.5 (−0.3, 3.3), 296 pairs	0.479 (p<0.001)

*QOLAD scored out or 60, ADRQL out of 100.

+ Reference is self rated QOL-AD and staff rated ADRQL respectively.

Although significantly correlated, staff and family informant ratings using the QoL-AD were about 7 points lower than self-ratings (mean difference −7.8, 95% CI −8.8, −6.7 for staff rated QoL-AD; and mean difference −7.2, 95% CI −8.5, −6.0 for family rated QoL-AD). There were no significant differences between staff and family informant rated QoL-AD (mean difference −0.5, 95% CI −1.5, 0.51) or ADRQL (Table 1).

Self-ratings of QoL were available for 226 PWD. Scores were lower among people who had been restrained, either documented (mean difference −3.9; 95% CI −6.0, −1.9) or observed (mean difference −4.1, 95% CI −7.0, −1.2), who had fallen in the last month (mean difference −2.2; 95% CI −4.4, −0.3), who reported pain (mean difference −3.5; 95%CI −5.3, −1.6) or had observed pain (mean difference −4.9, 95% CI −7.6, −2.3). Falls in the last month was the only factor consistently and significantly associated with informant ratings of QoL as well as self rating (Table 2). In contrast to self ratings, lower informant ratings of QoL were consistently apparent among people with severe cognitive impairment, neuropsychiatric symptoms or care giver distress. GP review, case conferencing, documented restraint, a secure perimeter, and hospital presentations also tended to be consistently (but not always significantly) associated with lower informant QoL ratings by family and staff informants. (Table 2).

**Table 2:** Factors associated with rating of quality of life among 226 residents able to self rate: mean differences (95%CI) relative to comparison group in unadjusted* quality of life scores associated with predictor variables (e.g., age >86 years compared with age <86 years, male cf female).

Variable	QOLAD Self	QOLAD Staff	QOLAD NOK	ARDOL Staff	ARDOL NOK
Age >86 years	0.08 (−1.47, 1.63)	−0.59 (−2.58, 1.40)	**2.67 (0.34, 5.01)**	0.79 (−3.09, 4.66)	1.05 (−2.49, 4.58)
Male Gender	1.17 (−0.59, 2.94)	1.20 (−1.08, 3.48)	2.51 (−0.29, 5.31)	−0.89 (−5.35, 3.57)	−0.24 (−4.47, 3.99)
MMSE <10	−1.27 (−3.35, 0.81)	**−3.19 (−5.91, −0.47)**	**−4.47 (−7.74, −1.20)**	**−6.90 (−12.06, −1.74)**	**−5.37 (−10.33, −0.41)**
GP review	−0.64 (−2.42, 1.15)	−0.52 (−2.85, 1.80)	**−3.04 (−5.73, −0.36)**	−0.66 (−5.12, 3.80)	−0.23 (−4.31, 3.86)
CMA	−0.08 (−2.07, 1.90)	1.89 (−0.60, 4.39)	−1.06 (−3.95, 1.83)	3.96 (−0.92, 8.84)	0.05 (−4.41, 4.51)
Case conference	−1.43 (−3.22, 0.36)	−0.22 (−2.57, 2.13)	−2.09 (−4.84, 0.66)	**−5.96 (−10.27, −1.64)**	−2.01 (−6.16, 2.13)
Pain assessment	−0.60 (−2.27, 1.06)	0.43 (−1.73, 2.58)	1.50 (−1.14, 4.13)	−2.63 (−6.73, 1.48)	2.16 (−1.73, 6.04)
Restraint documented	**−3.94 (−5.99, −1.89)**	−2.48 (−5.19, 0.22)	**−3.38 (−6.66, −0.10)**	−1.65 (−6.94, 3.65)	−0.08 (−5.05, 4.88)
Restraint observed	**−4.08 (−7.00, −1.16)**	−3.07 (−6.70, 0.56)	**−6.21 (−10.80, −1.62)**	2.90 (−4.41, 10.21)	−1.96 (−8.83, 4.91)
Perimeter secure	0.04 (−1.79, 1.86)	−0.48 (−2.83, 1.88)	−1.85 (−4.65, 0.94)	**−7.03 (−11.20, −2.85)**	**−7.99 (−11.59, −4.39)**
Hospital Presentation	−1.06 (−3.96, 1.84)	**−5.75 (−9.47, −2.03)**	−4.80 (−9.63, 0.04)	**−9.26 (−16.41, −2.12)**	−2.06 (−9.12, 5.00)
Falls (last month)	**−2.32 (−4.34, −0.30)**	**−5.15 (−7.61, −2.70)**	**−4.21 (−7.30, −1.11)**	**−5.56 (−10.59, −0.53)**	**−4.95 (−9.62, −0.29)**
>10 medications	−1.50 (−3.02, 0.03)	−0.65 (−2.62, 1.33)	0.11 (−2.29, 2.50)	2.04 (−1.77, 5.84)	1.60 (−1.97, 5.16)
Weight >60 kg	0.88 (−0.64, 2.40)	1.34 (−0.60, 3.28)	0.34 (−2.03, 2.71)	**3.82 (0.03, 7.61)**	0.70 (−2.87, 4.26)
Decreasing weight	1.05 (−0.59, 2.69)	1.10 (−1.06, 3.25)	**3.57 (1.01, 6.13)**	−0.28 (−4.51, 3.94)	−0.27 (−4.18, 3.63)
Pain (RVBPI)	**−3.46 (−5.34, −1.57)**	0.08 (−2.34, 2.50)	**4.58 (1.69, 7.46)**	3.71 (−1.06, 8.47)	6.43 (2.13, 10.73)
Pain (PAIN-AD>1)	**−4.93 (−7.59, −2.26)**	−1.60 (−4.95, 1.76)	0.55 (−3.81, 4.92)	−1.42 (−8.29, 5.46)	0.68 (−5.66, 7.03)
NPI >14	−1.50 (−3.03, 0.03)	**−5.36 (−7.21, −3.52)**	**−3.72 (−6.07, −1.37)**	**−16.56 (−19.74, −13.38)**	**−7.18 (−10.60, −3.76)**
Staff distress	−1.47 (−3.03, 0.09)	**−3.76 (−5.71, −1.82)**	**−3.00 (−5.39, −0.61)**	**−10.54 (−14.17, −6.91)**	**−4.64 (−8.18, −1.10)**

CMA  =  comprehensive medical assessment, RVBPI  =  Residents verbal brief pain inventory, NPI =  neuro-psychiatric inventory-NH.

*QOLAD scored out or 60, ADRQL out of 10.

In the regression models, significant univariate associations with self rated QoL-AD were found for restraint (documented and observed), number of medications and falls in the prior month, pain (reported and observed), and NPI-NH (both overall score and staff distress score). However, of these factors, only documented restraints (*B* = −0.232), reported pain (*B* = −0.251) and NPI-NH score (*B* = −0.158) remained independently associated with self rated QoL-AD in the final parsimonious multivariate model (adjusted R^2^ = 0.128).

Significant univariate associations were found between staff rated ADRQL and MMSE score, case conferencing, restraint (documented and observed), a secure perimeter, hospitalisation in the previous month, number of falls in the previous months, weight, observed pain and NPI scores (overall, and care-giver distress score). In the final parsimonious multivariate model MMSE score (*B* = 0.307), case conferencing (*B* = −0.092), hospitalization in the previous month (*B* = −0.162) and NPI-NH score (*B* = −0.523) were independently and significantly associated with staff rated ADRQL (adjusted R^2^ = 0.467).

## Discussion

### Main findings

These data show that most people with dementia living in RCF can rate their own QoL, and that informant ratings of QoL substantially underestimate self-ratings. These data support the validity of self-rating of QoL by PWD, given that self-ratings were associated with objectively observed factors (pain and restraint) that are expected to be associated with poorer quality of life. Our data also suggest that people with dementia can be engaged with to assess reasons for distress. It was uncommon for a staff informant not to be available, and we did not find evidence that family informant ratings more closely approximated self-ratings than staff informant ratings.

Our data confirm that some factors associated with informant ratings of QoL (such as severe cognitive impairment) do not appear to be significantly associated with self ratings of QoL. Conversely, some factors associated with self rating of QoL (such as reported pain) do not appear to be associated with informant ratings. A poor association of informant report with self report and direct observation has also been described in the assessment of pain in the RCF setting.^17^ Our data emphasise that the perspectives of PWD and their informants are not necessarily congruent and that factors affecting the relationship between informants and PWD may influence informants' perceptions of the QoL of PWD.

The tendency for factors such as case conferencing and GP review to be associated with lower informant QoL ratings was unexpected. This could represent an error due to chance, or confounding due to active clinical problems or recognition of unmet needs. Alternatively individuals who are the most complex, or are perceived as “troublesome”, may be identified for case conferencing. Case conferencing may also lead to unmet needs being identified, thereby influencing QoL ratings adversely.

### Strengths and limitations

Our data set was large and included process measures that may be markers of higher quality care (such as documentation of pain assessment) as well as resident focused quality of life measures. For complex variables, such as pain, we measured multiple candidate predictor variables comprising both observational and self report tools in an attempt to optimize measurement of these factors in this population. However we did not specifically collect data on several factors which may be relevant (such as functional status and frequency of family visits). Interpretation of these data is limited as we did not validate self ratings of QoL with other measures of personal experience, nor did we establish the test-retest reliability of self ratings. In addition our approach was chosen to provide information regarding the magnitude of differences in QoL ratings by patients, family informants and RCF staff associated with predictor variables categorized in a clinically meaningful way. Moreover, our sample of participating RCF is likely to be subject to a volunteer bias. Finally, we did not validate the quality of care provided, which may be an important consideration given that factors such as use of restraints and the incidence of falls may be influenced by facility and staff related factors. It is not clear whether the associations that we observed are explained by these staff and facility related factors.

### Conclusion

These data have important clinical implications, indicating that clinicians should give importance to the person with dementia's rating of quality of life. Informant ratings of QoL (by both staff and family carers) should be interpreted cautiously, as they do not directly represent patients' perceptions, needs and aspirations. Informant ratings underestimate self ratings of QoL of PWD and appear to be associated with factors (such as severity of cognitive impairment) which are not associated with self ratings. Although gathering QoL data from multiple perspectives may be informative, our data suggest that quantitative assessment of QoL will not necessarily be enhanced by collecting data from both staff and family informants. Many (well meaning) care giving situations impose limitations on people with cognitive impairments.[20] Thus clinicians and care-givers need to be cognizant of potential biases when considering their opinion of the QoL of PWD. Prompting staff and families to consider these different perspectives may be an important aspect of improving care for people with dementia living in residential facilities.
